# All the small things: How virus‐like particles and liposomes modulate allergic immune responses

**DOI:** 10.1002/eji.201847810

**Published:** 2019-12-15

**Authors:** Bernhard Kratzer, Sandra Hofer, Maja Zabel, Winfried F. Pickl

**Affiliations:** ^1^ Institute of Immunology Center for Pathophysiology Infectiology and Immunology Medical University of Vienna Austria

**Keywords:** allergy, immunotherapy, neutralizing antibodies, liposomes, virus‐like particles

## Abstract

Recent years have seen a dramatic increase in the range of applications of virus‐like nanoparticle (VNP)‐ and liposome‐based antigen delivery systems for the treatment of allergies. These platforms rely on a growing number of inert virus‐backbones or distinct lipid formulations and intend to engage the host's innate and/or adaptive immune system by virtue of their co‐delivered immunogens. Due to their particulate nature, VNP and liposomal preparations are also capable of breaking tolerance against endogenous cytokines, Igs, and their receptors, allowing for the facile induction of anti‐cytokine, anti‐IgE, or anti‐FcεR antibodies in the host. We here discuss the “pros and cons” of inducing such neutralizing autoantibodies. Moreover, we cover another major theme of the last years, i.e., the engineering of non‐anaphylactogenic particles and the elucidation of the parameters relevant for the specific trafficking and processing of such particles in vivo. Finally, we put the various technical advances in VNP‐ and liposome‐research into (pre‐)clinical context by referring and critically discussing the relevant studies performed to treat allergic diseases.

## Graphical abstract

Virus‐like nanoparticles (VNP) and liposomes are innovative therapeutic platforms for the modulation of allergic immune responses. They variably deliver cues for innate‐, antigen‐, and cytokine‐receptors and thereby induce blocking antibodies (either against effector cytokines of allergens) and/or T cell tolerance.

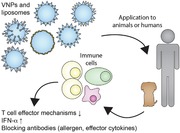

## Introduction

The human immune system is continuously exposed to foreign material, a large fraction of it encompassing microscopic to sub‐microscopic particles in the form of fungi, bacteria, or viruses 1. In fact, the formation of protocells and capsules, thus very simple particles (comparable to nowadays viruses and bacteria) enclosing genetic material and enabling simple biosynthetic reactions to take place with high efficiency within a spacially privileged environment, represents a process that is strongly connected with the beginnings of life 2. Similarly connected to early life forms was the threat to suffer from parasitism or fusion with such particles, i.e., the danger that one (usually lower) organism either starts to live at the expense of another (usually higher) organism or even fuses its genetic material with the latter 1.

Thus, it is not entirely surprising that the human immune system is especially capable of recognizing and reacting against compartmentalized foreign material in the form of microscopic or sub‐microscopic particles, because particle‐borne antigens may, at any time, represent a potential danger for the respective higher organism and strong barriers against fusion of different organisms have developed early on 3.

It is therefore conceivable that any foreign material, which is organized as a particle, even if non‐infectious, is seen by the human immune system with great(er) attention, worthwhile to be probed for its dignity, when compared to its soluble counterparts 4, 5. In fact, the particulate nature of non‐infectious particles containing potentially immunogenic proteins, such as tree and plant pollen, fungal spores, and house dust mite‐derived (fecal) particles, has been regarded as an important factor contributing to their sensitizing (IgE‐inducing) and allergic (disease‐promoting) potential 6, 7. Epidemiological studies further support the notion that also artificial nanoparticles, such as diesel exhaust particles, airborne carbon nanotubes (derived from uncontrolled wood burning), and microplastic may, at least, promote allergic sensitization 6, 7, 8, 9, 10, 11, 12, 13, 14, 15.

Given the large degree of attention the immune system is paying to the encounter with nanoparticles, a number of strategies based on nanoparticle technology and intended to induce desired or modulate undesired immune reactions for the benefit of patients have been developed 16, 17, 18, 19. Along those lines, virus‐like nanoparticles (VNP) but also liposomal preparations have been established and characterized recently 20. Paying tribute to the increasing incidence of allergies, which has reached 30% within our populations 21, this review will highlight the latest advances regarding nanoparticle‐based treatment strategies of allergic diseases explaining how virus‐like nanoparticles but also artificially synthetized liposomes may help to fight and/or protect from this widespread disease. VNP are derivatives of WT viruses, which are unable to fuse with target cells because they lack both Env (viral spike) proteins and an infectious viral genome or are derived of nonhuman pathogenic viruses. Until today, several anti‐infectious vaccines based on VNP technology have been approved for patient application 22, 23, which display excellent safety characteristics and elicit strong humoral and/or cellular immune responses 18, proving the efficacy of VNP‐based antigen delivery. In the following, we review particle‐based approaches intended to protect from or to treat already established allergic diseases.

## VNP‐based strategies for allergy treatment

**Figure 1:**
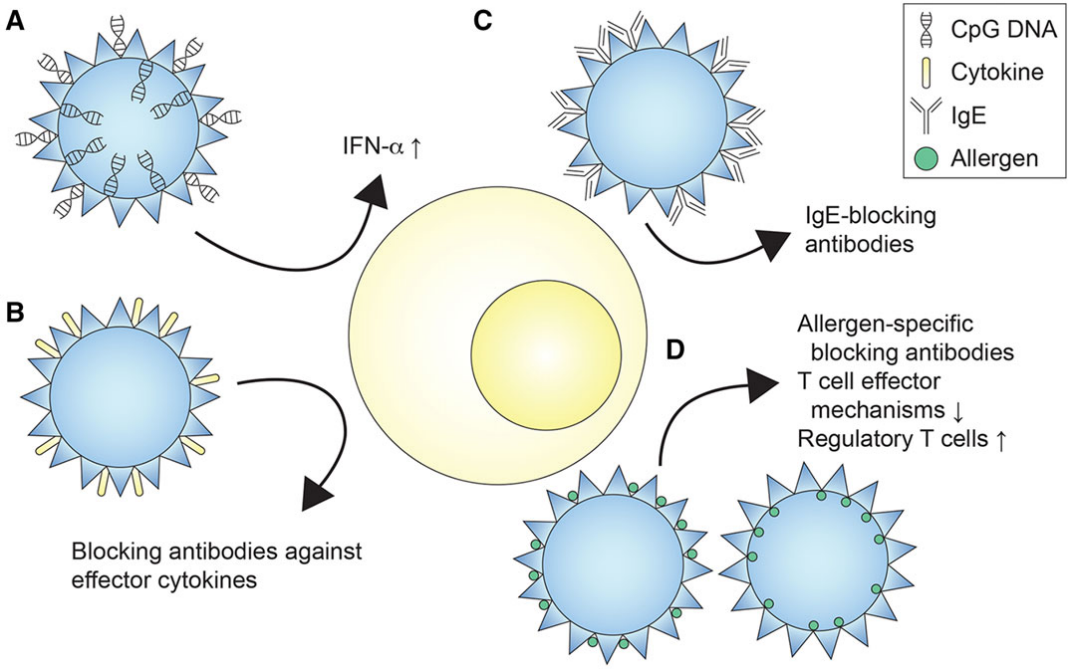
Different VNP‐based approaches for the modulation of allergen‐specific immune responses. Shown are VNP expressing (A) CpG DNA, (B) effector cytokines (IL‐4, IL‐5, IL‐13, IL‐23p40, IL‐31, IL‐33, TGF‐β, eotaxin), (C) human IgE or peptides thereof, (D) allergens as full‐length protein or in peptide form either expressed on the surface or shielded inside of particles. Induced mechanisms comprise the induction of allergen‐specific blocking antibodies, the modulation of T cell effector mechanisms, and the induction of regulatory T cells.

The different VNP‐based approaches aimed at the treatment of allergic diseases (Table 1) can be subsumed as follows: (i) VNP inducing antigen‐independent immunomodulation, e.g., by TLR ligands; VNP priming the production of neutralizing antibodies against (ii) effector cytokines of allergic immune reactions or (iii) allergen‐specific IgE and (iv) VNP eliciting allergen‐dependent immunomodulation (Fig. 1).

**Table 1:** Virus backbones used for the delivery of allergens or effector molecules important for allergic immune reactions

VNP ID*	Immunogen	Particle size (nm)	Reference
(i) VNP inducing antigen‐independent immunomodulation			
Qβ‐G10	CpG motif G10	30	27, 28, 29, 30
(ii) VNP priming the production of neutralizing antibodies against effector cytokines of allergic immune reactions			
CuMV	Equine IL‐5	30–40	42, 51
CuMV	Canine IL‐31	30–40	48
HBcAg	Mouse IL‐23p40	25	47
HBcAg	Mouse TGF‐β	25	50
HBcAg/HBcAg‐33	Mouse IL‐33	25	49
HBcAg/HBcAg‐A13	Mouse IL‐4‐Peptide	25	41
HBcAg/HbcAg‐LA	Mouse IL‐13 Peptide	25	44, 45, 46
Qβ	Mouse IL‐5 and mouse eotaxin	30	43
(iii) VNP priming the production of neutralizing antibodies against allergen‐specific IgE			
HBcAg split core	Human IgE peptide C3ε	30–34	79
HBcAg	Human CεmX peptide	30	80
HBsAg	Three hIgE peptides from the C3ε domain	22	78
Qβ	Two hIgE peptides of the C3ε domain	30	87
(iv) VNP eliciting allergen‐dependent immunomodulation			
CuMV	Fel d 1	30–40	94
Mo‐MLV	Art v 1	100	91
Mo‐MLV	Art v 1 peptide on MHC class II molecules	100	101
Qβ	Fel d 1	30	89, 90
Qβ	Der p 1	30	88
Qβ	HDM extract	30	92
Ty	Der p1	60	96, 97
Ty	Asp f 2 peptides	60	100
Ty	Asp f 2 and Asp f 3 peptides	60	99, 100

CuMV, cucumber mosaic virus; HBcAg, hepatitis B core antigen; HBsAg, hepatitis B surface antigen; Mo‐MLV, Moloney murine leukemia virus; Qβ, Qβ bacterial phage; Ty, Ty‐transposon from yeast *S. cerevisiae*.

### VNP inducing antigen‐independent immunomodulation

Antigen‐independent immunomodulation in the context of allergies comprises the ligation of pattern recognition receptors, such as TLRs, known to primarily induce Th1 immune responses 24. The possible advantages of VNP‐based, antigen‐independent, immune‐modifying treatment approaches may be severalfold. For instance, this therapy could be applied to allergic individuals without exactly knowing their sensitization profile 21. Moreover, the therapy might be well suited for individuals sensitized against complex allergen sources, in which the identification of the primary sensitizer and thus the best target for AIT might be difficult. For example, patients suffering from HDM allergy may be sensitized against a whole collection of HDM‐derived allergens, not all of them necessarily present in sufficient amounts in the different commercially available and currently approved extracts for AIT 25.

Along those lines, Qβ particles 26, consisting of the bacteriophage Qβ virus shell and containing the TLR9 A‐type CpG motif G10, a strong IFN‐α but not IL‐12 inducer in humans, have been evaluated in clinical trials in patients suffering from rhinoconjunctivitis and asthma 27, 28, 29, 30. In fact, six weekly injections with Qβ‐G10 VNP ameliorated symptoms of patients suffering from house dust mite‐induced allergic rhinitis, resulting in significantly reduced medication use and a tenfold increase in the tolerized conjunctival provocation dose 30. However, the authors pointed out in their study that HDM represent an ubiquitous allergen source and the possibility that the patients were exposed to (minimal) amounts of HDM‐allergen while receiving the vaccine could not be ruled out completely. Thus, an antigen‐dependent component contributing to vaccine efficacy could not be entirely excluded in that study 31. In a subsequent study, patients suffering from mild‐to‐moderate persistent allergic asthma against one aeroallergen were treated with Qβ‐G10 particles. Treated patients presented with lower asthma symptom scores and significant stabilization of their lung function (FEV_1_) when compared to the placebo‐treated control group upon steroid withdrawal 27. However, in a DBPC phase 2b study performed with patients suffering from moderate‐to‐severe asthma and treated with inhaled steroids with or without LABA, vaccination with Qβ‐G10 particles showed no additional benefit over placebo treatment, questioning the efficacy of the antigen‐independent immune‐modifying treatment with Qβ‐G10 particles, at least in that patient group 32. Since then, no further studies with Qβ‐G10 particles have been performed.

Taken together, antigen‐independent immune‐modifying therapies may have clinical benefits in selected groups of individuals, however, careful elucidation of the immunological mechanisms and pathways triggered by them will be required to better understand the longevity of the induced changes 27, 28, 29, 30 and their potential side effects 28.

### VNP priming the production of neutralizing antibodies against effector cytokines of allergic immune reactions

This approach aims at the active induction of neutralizing autoantibodies against effector cytokines in allergic diseases. The active immunization approach follows a similar logic as passive immunization with biologics, which, in the recent years, has become an established treatment for severe cases of, e.g., allergic asthma by targeting effector molecules critically involved in the allergen‐specific immune response like IL‐4, IL‐5, or IL‐13 33, 34, 35, 36, 37, 38. However, compared to the high treatment costs and efforts (repeated injections) associated with passive immunization, active immunization against immune effector molecules might be advantageous, because it could represent a more cost‐effective therapy with the potential to induce a durable, long‐term, and polyclonal response against the targeted molecules. Moreover, the polyclonality of the active immune responses will reduce the likelihood of the induction of neutralizing antidrug antibodies (ADA), which otherwise represent major impediments of therapies based on monoclonal biologics 39, 40. In the following section, we contrast preclinical data of active immunization trials, which have exclusively been performed in experimental or companion animals, with results of human clinical trials obtained upon passive immunization with antibodies targeting the respective cytokines.

In the past, a number of type 2 effector cytokines, either full‐length or peptides thereof, such as IL‐4 41, IL‐5 alone 42, or in combination with eotaxin 43, IL‐13 44, 45, 46, IL‐23p40 47, IL‐31 48, IL‐33 49, and TGF‐β 50, have been chemically conjugated to or expressed on the surface of VNP followed by evaluation of their immunogenicity and their impact on different facets of allergic diseases in experimental animals in vivo. In fact, most VNP versions actively induced anti‐cytokine antibodies (except TGF‐β VNP) 50, and reduced hallmarks of allergic diseases in preclinical models. Interestingly, none of these studies found signs of therapy‐associated adverse reactions in the preclinical models conducted 41, 42, 43, 44, 45, 46, 47, 48, 49, 51. However, the microbiologic burden of inbred mice housed in a clean or even specific pathogen free (SPF) environment is certainly not comparable to outbred populations living under unconstrained conditions. Vaccination studies undertaken, for instance, with horses, dogs, or cats are therefore certainly better suited to draw relevant conclusions as to the safety of actively inducing anti‐cytokine antibodies, since they usually remain exposed to their natural habitat when undergoing VNP‐based therapy for allergy. Moreover, similar to human individuals and unlike mice these animals naturally suffer from allergic diseases affecting target organs, which also have relevance for humans, such as the skin and the respiratory tract 52. In one of the first studies intended to induce autoantibodies against an endogenous cytokine, Ma et al. induced neutralizing IL‐4 antibodies in mice, which protected them from subsequent sensitization with OVA as reflected by reductions in OVA‐specific serum IgE, eosinophil numbers in BALF, goblet cell hyperplasia, tissue inflammation, and methacholine‐induced airway hyper‐reactivity. Apart from its ability to induce Ig class switch recombination to IgE, IL‐4 is also important for multiple other aspects of maintaining immune homeostasis 41. In fact, IL‐4 reduces Th1 inflammation 53, facilitates B cell‐mediated antigen presentation by upregulating MHC class II molecules 54, and increases adhesion of T cells to endothelial cells 55. Despite these important tasks, no side effects upon permanent neutralization of IL‐4 were detectable in the mouse model of OVA allergy investigated. This is in contrast to passive immunotherapy performed with the anti‐IL‐4/IL‐13 antibody dupilumab in human patients, which seems to be associated with an increased risk for the development of conjunctivitis 56. Recently, vaccination with cucumber mosaic virus (CuMV)‐based VNP expressing equine IL‐5 showed promising results in Icelandic horses suffering from chronic allergic dermatitis caused by insect bites and consisting of a mixed type I/type IV immune reaction 57 with prominent eosinophil infiltration 42. Similarly effective were combined immunizations with Qβ particles chemically conjugated with either eotaxin or IL‐5, aiming at the reduction of eosinophil recruitment and expansion in a mouse model of OVA allergy 43. Despite the important biological roles of IL‐5 for the defense against (reinfections with) helminths 58, no impact of anti‐IL‐5 antibodies on the natural worm burden in horses vaccinated against IL‐5 was evident 42, 51. Moreover, tolerance against IL‐13 has been successfully broken in vivo upon vaccination of mice with VNP, formed by genetic fusion of an IL‐13 peptide to the hepatitis B core antigen (HBcAg) 44, 45, 46. IL‐13‐HBcAg VNP attenuated airway inflammation and remodeling and proved to be effective even when applied via the mucosal route 45. Similar to the active immunization against IL‐13 in mice, passive administration of anti‐IL‐13 mAbs (lebrikizumab) in human clinical trials reduced asthma exacerbations and improved lung function (FEV_1_) especially in those patients presenting with high pretreatment periostin levels 59, 60. The IL‐23/IL‐17 signaling pathway has been identified as another important signaling axis, with relevance for allergic diseases such as atopic dermatitis and asthma in which Th17 cells play a central role 61, 62. Accordingly, Guan and colleagues fused a nonapeptide of the IL‐23p40 subunit (which is identical to IL‐12p40) to the HBcAg. The recombinantly produced IL‐23p40‐HBcAg fusion protein spontaneously assembled into VNP and was used to subcutaneously immunize experimental animals followed by their sensitization with OVA. Notably, vaccination with IL‐23p40‐HBcAg VNP significantly reduced total and OVA‐specific IgE titers, numbers of neutrophils and eosinophils in BALF as well as goblet cell hyperplasia and inflammation of lungs in mice 47. However, as the authors have shown, the approach of targeting IL‐23p40 intrinsically generated also anti‐IL‐12 autoantibodies, with the potential to weaken IL‐12‐dependent Th1 pathways, although no such activity was detectable during the observational period of their study 47. Moreover, targeting of IL‐31 in atopic dogs by active immunization with IL‐31 conjugated to CuMV containing a universal T cell epitope from *Tetanus* toxin TT830‐845 63 resulted in the induction of neutralizing IL‐31 antibodies, which significantly reduced the scratching behavior of animals in more than 80% of dogs immunized 48. For the same indication, an IL‐31 blocking antibody was licensed for human use recently, which, upon passive administration, caused no side effects 64, 65, 66. Another important factor, mostly produced by and released from epithelial cells is the alarmin IL‐33, which is supposed to be one of the most important initiators of type 2 immune reactions 67. Targeting of IL‐33 by active immunization with IL‐33‐HBcAg‐based VNP has been shown to reduce the severity of allergic asthma in a mouse model and might be an interesting therapy option to neutralize the very early effects of epithelial damage on both innate and adaptive immunity such as priming of Th2 development and activation of ILC2 49. However, the diverse and important roles of IL‐33 in other organ systems (e.g., gut, etc.) and during several important defense mechanisms (e.g., against worms) need to be taken into account before a final conclusion as to the safety and applicability of inducing anti‐IL‐33 immunity can be made.

### General considerations for the induction of auto antibodies

Active induction of anti‐cytokine antibodies represents a powerful tool and a possible alternative to the passive application of mAbs neutralizing the very same cytokines, because the method of breaking self‐tolerance against cytokines seems to be effective, cost saving, and requiring only a limited number of vaccine doses 48. As appealing as the possibility of inducing blocking autoantibodies against pathognomonic effector cytokines may be, it is also apparent that the breakage of tolerance might potentially lead to adverse events, including undesired autoimmune phenomena or specific immunodeficiency, which, once induced, may be hard to reverse. Such risks may include the re‐activation of latent infections, similar to the ones observed upon passive immunization with TNF‐α inhibitors leading to the activation of latent tuberculosis 68, or have a permanent impact on wound healing and tissue remodeling, e.g., aggravating episodes of myocardial infarction as observed in IL‐13 KO mice 69. Vaccine‐induced B cell memory cells directed against bodily constituents might be hard to remove, the same accounts for long‐lived plasma cells once established. This risk is in clear contrast to the passively administered mAbs, which administration can be stopped at any time and then become catabolized and disappear from the organism after three to six half‐lives 70. Another potential issue of the in vivo‐induced autoantibody responses against cytokines is the possibility that such autoantibodies instead of mitigating might rather potentiate the function of the targeted cytokines, especially when they start to react with restricted epitopes of the respective cytokines 71. For instance, monoclonal anti‐IL‐2 antibodies, upon binding to their target, not only prolong the half‐life and thus the overall activity of IL‐2, but also preferentially target the complexed IL‐2 to either the low or high‐affinity IL‐2R, expanding either CD4^+^ T regulatory or CD8^+^ cytotoxic T cells 71. Thus, it is essential to make sure that active immunization with cytokine‐VNPs induces a polyclonal, neutralizing antibody response from the beginning.

Apart from the potential danger that cytokine‐VNP vaccine preparations might actually potentiate the function of the targeted cytokine, it has also been clearly demonstrated that particle borne cytokines, such as GM‐CSF in combination with IL‐4 72, but also IL‐2 73, perfectly retain their biological activity when tethered to the surface of VNP, which again, due to the very function of the respective cytokine, might lead to adverse effects upon in vivo application (e.g., cytokine storm, vascular leakage syndrome, etc.) 74, 75, 76, 77.

### VNP priming the production of neutralizing/blocking antibodies against IgE or FcεRs

Some of the most powerful approaches to interfere with IgE‐associated allergies are those that target IgE, their specific receptors (FcεRs), and/or IgE‐producing B cells/plasma cells directly 78, 79, 80. These treatment modalities intend to remove IgE‐producing cells themselves or to neutralize the effector function of IgE. The anti‐IgE mAb omalizumab (Xolair^R^) is in clinical use for the treatment of asthma 36 since 2005 and for the treatment of chronic idiopathic or spontaneous urticaria 81 since 2014. Omalizumab specifically binds to the C3ε region of IgE and thus neutralizes its binding to FcεRs 82 and dampens Th2 inflammation 83; however, similar to other biologics, omalizumab has to be applied systemically (i.e., subcutaneously) in intervals of 2–4 weeks. Accordingly, several studies have investigated the possibility to break tolerance against IgE or FcεRs by inducing respective autoantibodies/blocking antibodies against them. All experimental systems described in the following took advantage of human IgE/FcεR to induce blocking antibodies/break tolerance in the murine system. The first approach has been developed by Peng et al., who conjugated three IgE‐derived peptides corresponding to the human IgE receptor‐binding site of IgE to the HBsAg as the carrier 78. Notably, the antibodies induced in rats blocked the binding of soluble rat IgE to rat FcεR and also downregulated rat serum IgE‐antibody levels; however, they did not react with FcεR‐bound rat IgE, demonstrating the safety (non‐anaphylactogenicity) of the induced autoantibodies. The split core technology 84, which allows for the expression of structural epitopes on the surface of hepatitis B particles (HBcAg), was used to generate blocking antibodies, which specifically target the receptor‐contacting site of the human C3ε domain of IgE 79. Immunization of mice with such IgE‐epitope‐HBcAg particles induced high‐titer (>1:36 000) anti‐human IgE antibodies 79. Another study showed that human IgE can even be targeted in its transmembrane form, as expressed on human IgE‐producing B cells/plasma cells, and without simultaneously targeting receptor‐bound IgE. An elegant demonstration of such selective targeting of surface‐expressed IgE on B cells/plasma cells has come from Lin et al., who selected an epitope of IgE (CεmX), which is exclusively expressed on the long isoform of cell surface‐expressed human IgE. It consists of 52 amino acids and is located between the C4ε domain of IgE and its C‐terminal membrane‐anchor peptide 85, 86. Notably, the induced blocking anti‐IgE antibodies were able to elicit antibody‐dependent cellular cytotoxicity (ADCC) against IgE‐expressing mouse myeloma cells as well as the Burkitt's lymphoma‐derived Ramos cell line 80. Recently, Akache et al. used Qβ VNP, conjugated with two different peptides from the human Cε3 domain of IgE to activate the innate immune system (in humans and mice) and induce blocking anti‐IgE antibodies in mice. Within their study, they elegantly demonstrated that the resulting immune response of human PBMCs in vitro is similar to the action of TLR7 (reacting with bacterial RNA) leading to the induction of IFN‐α, which can be blocked with chloroquine. In vivo, in mice (upon 3× i.m. injection with 4 weeks interval), such particles induced high titers of blocking anti‐IgE antibodies, who's production was strongly reduced in TLR7 KO mice. For anti‐IgE vaccines mixed with alum or CpG/alum, no reduction in TLR7 KO mice was observed. This study demonstrated the importance of innate danger signals harbored within VNP‐derived vaccines, which either can be derived from the producer organism itself (e.g., bacterial RNA) or become artificially admixed to the VNP‐based vaccine (alum, CpG DNA, etc.) 87.

Although seemingly attractive, no clinical studies targeting IgE or FcεR by active (VNP‐based) immunization have been registered as of today.

### VNP eliciting allergen‐dependent immunomodulation

The third VNP‐based approach targets the culprit allergens directly and aims at the induction of allergen‐specific T cell tolerance and/or the induction of blocking antibodies 21. Surface attachment of full‐length allergen or B cell epitopes thereof to VNPs opens the opportunity for the induction of blocking antibodies. However, this strategy also increases the risk for adverse reactions in already sensitized individuals, since allergen‐VNP might lead to IgE cross‐linking on effector cells resulting in mediator release and anaphylaxis. Until today, only a small collection of full‐length allergens have been attached/targeted to the surface of VNP and subsequently evaluated, which include the major house dust mite allergen Der p 1 88, the major cat dander allergen Fel d 1 89, 90, and the major mugwort pollen allergen Art v 1 91. Interestingly, both Engeroff et al. 89 and Kratzer et al. 91 could demonstrate that allergens displayed on the surface of VNP induced much weaker degranulation of IgE‐sensitized effector cells when compared to equimolar concentrations of the respective soluble allergens (Fel d 1 and Art v 1) 89, 91. From the above it can be argued that the mere cell surface association of an otherwise soluble allergen might render it hypoallergenic. So far, no clinical trials with VNP expressing allergen on their surface have been conducted in allergic individuals to prove these in vitro observations. However, Qβ‐G10 particles have been used as an adjuvant together with HDM allergens and clinically evaluated. These studies revealed that Qβ‐G10 particles were well‐tolerated and induced almost complete tolerance to the co‐administered allergens 92. Morover, Qβ‐Der p 1 VNP were evaluated in a safety phase I clinical trial by Kündig et al., which revealed strong induction of both, Der p 1‐ and Qβ (backbone)‐specific antibodies in healthy volunteers 88. Moreover, Qβ‐VNP decorated with the cat allergen Fel d 1 efficiently induced blocking antibodies that prevented sensitized mice, in an IL‐10 independent manner from active systemic anaphylaxis upon i.v. challenge with soluble rFel d 1 90. Recently, CuMV expressing Fel d 1 and containing the tetanus toxin–derived universal T cell helper epitope TT830‐843 have been devised and applied in cats to induce autoantibodies against their major cat allergen Fel d 1 93, 94. The induced blocking autoantibodies displayed high affinity for Fel d 1 and reduced Fel d 1‐levels systemically, i.e., also in tear fluid of animals, which, apart from saliva, is a rich source of that secretoglobulin 93, 94

Since full‐length allergens introduced systemically might induce anaphylaxis in sensitized individuals, alternative strategies restricted to T or B cell epitopes have been developed. Along those lines, the p1 protein of the yeast retrotransposon Ty, which has been shown to spontaneously assemble into VNP previously 95, has been conjugated with T cell epitopes of the mite allergen Der p 1 to devise Der p 1 Ty‐VNP, which were used to immunize mice intraperitoneally. Using that strategy, Harris and Hirschberg 96, 97 were able to induce allergen‐specific CD4^+^ T cell responses, which were Der p 1_111–139_ peptide specific 97 and abrogated allergen‐specific IL‐5 secretion 96 upon restimulation with Der p 1 peptide. In a similar vein, conjugation of the major T cell epitope of the *Aspergillus fumigatus* allergen Asp f to yeast p1 protein, known for its spontaneous assembly into VNP 98, 99, downregulated both, allergen‐specific T cell responses and allergen‐specific serum IgE, IgG_2a_, and Ig_G3_, but not IgG_1_ and IgG_2b_ levels 100. A slightly different approach was taken by Leb et al., who decorated MoMLV‐based VNP with HLA class II molecules already displaying the immunodominant epitope of the major mugwort pollen allergen Art v 1 in the presence or absence of distinct co‐stimulatory molecules 101. Allergen‐specific T cells incubated with such HLA/peptide‐VNP in the absence of co‐stimulators induced T cell anergy 102, 103, while HLA/peptide‐VNP co‐expressing CD58 prompted the differentiation of a unique T cell phenotype characterized by the production of IFN‐γ and IL‐10 101, commonly referred to as Tr1 cells 104. For these reasons, it appeared likely that, in principle, modulation with the help of VNP the allergen‐specific immune responses at the level of T cell activation could be successful. To circumvent the necessity for the display of a collection of HLA/peptide combinations on VNP in a patient‐tailored way for the treatment of allergic individuals, Kratzer et al. explored a clever alternative strategy by expressing full‐length allergens in a non‐anaphylactic and non‐IgE inducing form, by encapsulating them within MoMLV VNP 105. For that purpose, they relied on the full‐length major mugwort pollen allergen Art v 1 fused to the MoMLV matrix protein p15, which targets any fusion partner, so also the allergen, to the inside of the MoMLV VNP envelope. In a new mouse model of mugwort allergy 105, these authors could show that such VNP preparations are non‐anaphylactogenic and non‐sensitizing (IgE inducing) but instead able to induce tolerance if applied intranasally 91. To corroborate these finding, the authors plan to screen more patient sera and also plan to perform basophil activation tests with whole blood of mugwort allergic individuals.

The major attraction of allergen‐specific approaches for the prevention and treatment of allergies is their precise targeting of the molecular cause of the disease, i.e., the aberrant immune responses against the culprit allergen(s) without further affecting unrelated immune responses. Possible disadvantages of such technology lie, e.g., in the potentially imprecise targeting of the VNP delivered systemically to the “wrong,” immunoactivating APC and/or the instability of the enveloped viral particle, which, upon undesired disassembly would release full‐length allergens that may lead to effector cell activation and anaphylaxis. 89, 91. However, these potential caveats can be omitted by using strict T cell epitopes of major allergens, which are otherwise unable to crosslink IgE on allergen‐specific B cells or on sensitized effector cells 100.

Taken together, VNP‐based strategies for allergen‐dependent immunomodulation represent a powerful method for the efficient engagement of both the innate and adaptive immune system. Importantly, already early studies have shown that, in principle, vaccination with VNP represents a safe procedure 106. Thus, one of the challenges of the future will be to select the virus backbones most suitable for the different applications, i.e., induction of blocking antibodies and/or T cell tolerance, breaking of tolerance against endogenous effector molecules, activation of innate immune cells, etc.

## Liposomes for the modulation of allergic immune response

Recently, substantial progress has also been made toward the development of synthetic, lipid enveloped nanoparticles, also referred to as liposomes 107, 108. Liposomes are mostly based on phospholipid preparations, which spontaneously form mono‐ or multilayers of spherically shaped particles of rather heterogeneous size ranging from 50 to 450 nm 109, 110. In practice, protein antigens (and allergens) can either be attached to the outer surface of liposomes by chemical conjugation or may be encapsulated within or embedded in‐between the lumen of the lipid bi‐layer 111.

Several examples have emerged of the use of liposomes to modulate allergic immune responses (Table 2). They encompass allergen‐specific and ‐nonspecific treatment modalities with differently sized and charged liposome compositions 110, 111. For example, Inoh et al. investigated the influence of liposomes consisting of cationic 1,2‐dioleoyl‐sn‐glycero‐3‐phosphatidylethanolamine (DOPE) and cholesteryl‐3b‐carboxyamidoethylene‐*N*‐hydroxyethylamine (OH‐Chol) lacking allergens on mast cell activation in vitro and in vivo by monitoring allergic reactions with a focus on vascular permeability. Notably, even after antigen‐induced crosslinking of FcεRI, both the degranulation reactions of mast cells and vascular protein leakage were significantly reduced after pretreatment with such cationic liposomes 112.

**Table 2:** Liposomal preparations used for the delivery of allergens

Liposome composition*	Molar ratio of lipids	Immunogen	Particle size (µm)	Reference
DOPE: OH‐Chol	2:3	None	0.479	112
dipalmitoyl‐PC: Chol: mannotriose‐dipalmitoyl‐PE coated with oligomannose	10:10:1	OVA	1	113
myristoyl DMPC: DMPE:DMPG:Chol	4:3:2:7	OVA surface linked	0.2412	118
palmitoyl DMPC: DMPE:DMPG:Chol	4:3:2:7	OVA surface linked	0.2495	118
stearoyl DMPC:DMPE:DMPG:Chol	4:3:2:7	OVA surface linked	0.2373	118
oleoyl DMPC:DMPE:DMPG:Chol	4:3:2:7	OVA surface linked	0.2313	118
PC:PS:Chol	1:1:2	OVA	not tested	121
PC:PA:Chol	1:1:2	OVA	not tested	121
DDAB: PC: Chol		Fel d 1	3.5–5.4	125
Lipoid‐S‐100 PC: Chol: DDAB	2:1:1	Der p1, Der p2	1‐4.5	126
PC:Chol:DDAB	1:1:2	T289‐Per a 9, T167‐Per a 9, Per a 9	2‐5.7	128
OH‐Chol: DOPE	2:3	Alpha‐galactosylceramide with OVA	275	129
egg PC: L‐a‐dimyristryl phosphatidic acid: Chol	5:1:4	OVA DNA	nt	132
PC: 1,2‐dioleoyl‐3‐ trimethylammonium‐propane chloride salt: DOPE	9:1:1	OVA and CpG ODN 1826	130‐260	130

DOPE, 1,2‐dioleoyl‐sn‐glycero‐3‐phospha‐tidylethanolamine; OH‐Chol, cholesteryl‐3b‐carboxyamidoethylene‐*N*‐hydroxyethylamine; DMPC, dimiristoyl phosphatidyl choline; DMPE, dimiristoyl phosphatidyl ethanolamine; DMPG, dimiristoyl phosphatidyl glycerol; Chol, cholesterol; PC, phosphatidylcholine; PS, phosphatidylserine; PA, phosphatidic acid; DDAB, didecyldioctadecylammonium bromide.

To ensure proper processing of their contents, liposomes can also be efficiently targeted to immune cells of choice, e.g., to professional APCs, which can be accomplished by mannosylation 113, 114, 115. In one study, mannosylated liposomes containing the model allergen OVA induced suppression of specific serum IgE levels while OVA‐specific IgG_1_, IgG_2a_, and IgA production was increased after intranasal application of OVA in sensitized BALB/c mice 113.

Liposomes can also be used as platforms for the attachment of immunogenic proteins, against which a protective B cellular immune response should be induced 116, 117. Nakano et al. applied liposomes consisting of four different lipid components and surface‐linked OVA. All four variants induced IgE‐selective unresponsiveness, and, at the same time, IgG blocking antibodies against OVA 118. The highest anti‐OVA IgG antibody levels were obtained after immunization of mice with OVA‐liposomes made of unsaturated lipids, which likely increased the fluidity of the liposomal bi‐layer membrane and constituents located within that membrane 118. Notably, the omission of cholesterol decreased membrane fluidity and, in parallel, increased protective OVA‐specific IgG titers 119, indicating that the adjuvanticity of liposomes can be easily regulated by changing their membrane‐fluidity.

Liposome‐based delivery of allergens can also be used to induce allergen‐specific tolerance. For that purpose, charged liposomes that encapsulate allergens have been successfully explored as platform 116, 117, 120, 121. Introduced allergens either consisted of recombinant allergens or crude allergen extracts, which were variably combined with adjuvants embedded into the liposomes. Along those lines, Yotsumoto et al. demonstrated that liposomes containing OVA may induce the differentiation of IFN‐γ expressing, allergen‐specific Th1 cells in vitro and in vivo. Moreover, a higher liposomal content of phosphatidylserine (PS) induced both the secretion of IFN‐γ and IL‐12 while OVA‐specific IgE levels decreased. Notably, the PS‐dependent elaboration of IFN‐γ could be specifically blocked by the addition of soluble annexin V, which is a Ca^2+^‐dependent phospholipid binding protein with high affinity for PS 122, 123 initially described as a protein with strong anti‐coagulant activity 124. Liposomes containing increased levels of PS in their membranes may mimic apoptotic cells/cellular bodies that are well‐known “eat‐me‐signals” for phagocytes 121. Similar results were obtained with liposomes that either contained native Fel d 1 allergen or crude cat hair extract 125. Sensitized mice were treated with eight doses of allergen‐containing liposomes or extract alone and subsequently re‐challenged with allergen. Treatment with allergen‐containing liposomes but not extract alone decreased IgE levels, mucus production, and Th2 responses and increased Th1 and Treg levels 125 upon allergen re‐challenge, demonstrating the immunomodulating capability of liposomes.

Similar studies were performed with house dust mite sensitized mice, which were repeatedly treated with multilamellar liposomes applied intranasally. Liposomes consisting of lipoid‐S‐100 phosphatidylcholine (PC), didecyldioctadecylammonium bromide and cholesterol (C) either contained allergen extract, purified Der p 1, or Der p 2 (purified from extract) alone or in combination. Liposomes containing a single purified allergen induced expression of IL‐10, IL‐35, and TGF‐β in lung Th cells, while reduction of airway remodeling and Th2 responses as well as increases in Th1 cytokine expression were also induced by liposomes containing crude extract. However, the use of crude extracts is far from ideal due to potential impurities and poorly controllable allergen composition both affecting the degree of lung inflammation 126.

To circumvent such problems, Prangtaworn et al. used liposomes containing recombinant allergens derived from major cockroach allergens that they linked to T cell epitopes of IgG Fc, known to specifically expand regulatory T cells (Tregitopes) 127 for the induction of tolerance and the reduction of airway inflammation. Upon intranasal (seven times every other day) treatment of sensitized mice (intranasally to crude cockroach extract) the immune responses in the lungs were analyzed on day 63, together with blood and serum samples. Suppressor cytokines indicative of Treg activation, such as IL‐10, TGF‐β, and IL‐35 were expressed in the lungs after Tregitope289‐Per a 9 liposome treatment, whereas liposomes encapsulating Tregitope167‐Per a 9 liposomes revealed IL‐10 and TGF‐β expression only. Reduced type 2 inflammation was also obtained upon treatment with all Per a 9 containing liposomal preparations (recombinant Per a 9 alone and Tregitope Per a 9 liposomes); however, induction of Treg cells was exclusively observed when Tregitopes were present. Liposomes exclusively containing allergen were unable to induce a Treg signature but, instead, induced high levels of IFN‐γ production 128.

In another elegant study, liposomes containing the NKT cell stimulator α‐galactosylceramide as adjuvant were sublingually co‐administrated with soluble OVA to sensitized C57BL/6 mice. After re‐challenge with OVA, cervical LN CD4^+^ T cells elaborated decreased IL‐4, IL‐5, IL‐13, and IL‐17 on both the protein and mRNA level. In contrast, Th1 cytokine production was upregulated and paralleled by the production of increased levels of allergen‐specific IgG and reduced levels of IgE 129. Bal et al. further increased the immunomodulatory capacity of liposomes by combining the model allergen OVA with the TLR ligands PAM or CpG and encapsulated them into cationic liposomes 130. The forced TLR9 signaling led to the production of significantly higher levels of IgG antibodies against OVA and a Th1‐biased cellular response 130. Fusogenic liposomes were also used as vehicle 131 to deliver expressible cDNA of model allergens (OVA) and UV‐inactivated Sendai virus to achieve genetic immunization and thus influence the balance of Th cell subsets 132. By delivering OVA‐specific DNA within liposomes, Yoshikazu et al. were able to induce highly specific Th1 responses as well as OVA‐restricted CTL activity by engagement of the MHC class I antigen presentation pathway 132.

In summary, liposomes have the clear‐cut advantage that they can be assembled from individual, highly pure, chemical‐grade components, making the establishment of GMP for their standardized production simple and straight forward. In contrast, for liposomes to function properly in vivo, optimal formulations have to be found, guaranteeing their in vivo stability and their uptake and processing by APCs without eliciting undesired reactions against one of their principle components. For obvious reasons, such optimization steps are not needed for viruses that co‐evolved with mammals.

## How are nanoparticles recognized and taken‐up by immune cells?

**Figure 2:**
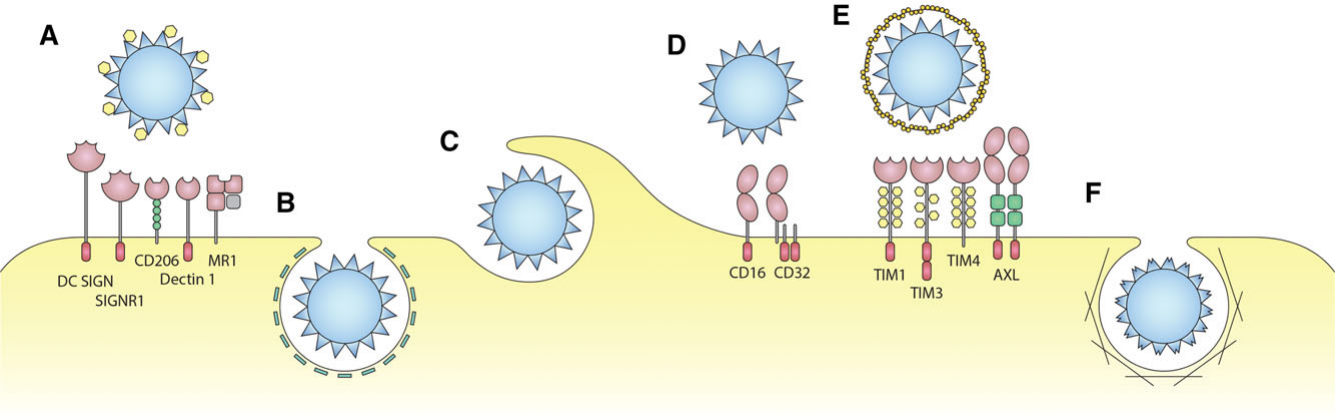
How DCs take up VNP. Shown are pathways dependent on (A) mannose receptors (DC SIGN, SIGNR 1, CD206, Dectin 1, MR 1), (B) clathrin‐dependent endocytosis (inhibited by heparin, chloropromazine or amiloride), (C) phagocytosis (inhibited by cytochalasin D), (D) Fc‐receptors (CD16, CD32), (E) phosphatidylserine‐specific receptors (TIM1, TIM3, TIM4, and AXL) and (F) macropinocytosis (dependent on actin).

Particulate material circulating in bodily fluids is considered potentially dangerous by the immune system for several reasons because particles might (i) represent infectious material, (ii) release toxins, (iii) seek to fuse its genetic material with bodily cells, and finally (iv) cause unintended (auto‐)immune reactions, e.g., against cellular debris such as apoptotic bodies displaying nucleic and ribonucleic acids, if not recognized and removed quickly and efficiently 133. In fact, if clearance of such endogenous particulate material is inefficient 134 or, alternatively, if such material is injected under experimental conditions 135, it will trigger autoimmunity. To prevent such potentially dangerous overload of the body with endogenous particulate material but also to protect from infectious agents, dendritic cells, monocytes and macrophages, all belonging to the mononuclear phagocyte system, very efficiently take up particulate material for degradation and/or presentation of its contents by different pathways 136. Critical parameters for the efficient uptake of particles are particle size 5, 137, 138 and/or the particles’ ability to specifically interact with scavenging surface receptors on phagocytes/APC (Fig. 2 and Table 3). No matter which receptor pathway takes the lead during VNP uptake and initiates an adaptive immune response, the mode of degradation and the quality of activation of the respective mononuclear phagocyte will decide whether antigen presentation will either be governed by immune activation or rather by tolerance induction 139. Specific analyses of APC upon interaction with particulate material may predict the induction of an active immune response. Such monitoring includes the determination of the APC activation marker molecules CD40, CD80, CD83, CD86 140, 141, and of the antigen‐presenting molecules itself, i.e., MHC class II 142, along with the release of inflammatory cytokines, such as IL‐6 or IL‐12 140, 143. The expression on VNP of preformed TCR ligands in the form of pMHC complexes along with classical co‐stimulatory molecules 144 or TLR‐ligands such as CpG oligonucleotides 90, 145 might lead to canonical activation of APCs and thus active (Th1) immunity against the co‐delivered allergen or peptides thereof. Moreover, biologically active activation and/or maturation factors of APCs attached to the VNP‐surface, such as GM‐CSF and IL‐4 might further shape the ensuing allergen‐specific immune response 17, 72, 146. However, for tolerance induction to occur, the activation of dendritic cells should rather be avoided and peptide‐presentation in a non‐inflammatory environment would be the preferred way 91.

**Table 3:** Receptors and pathways for the uptake of VNP and liposomes by APC

Uptake mechanism	Cell type	Involved receptors	Inhibitor	Evaluated VNP/viruses*	Reference
CD16/CD32 Fc‐receptors	DCs	CD16/CD32	CD16/CD32 double KO	HPV	159
Clathrin‐dependent endocytosis	HeLa cells		Heparin and chlorpromazine	AAV	158
	Mo‐DCs		Chlorpromazine	RHD	157
Macropinocytosis	Mo‐DCs, BM‐DCs		Amiloride	RHD	157
Mannose recognition	DCs, macrophages	DC SIGN, SIGNR1, CD206, Dectin 1, MR1	Mannose	RHD, Liposomes	117, 156
Phagocyotosis and Macropinocytis	Mo‐DCs, BM‐DCs		Cytochalasin D	RHD	157
Phosphatidylserine mediated uptake	DCs, macrophages	TIM1, TIM3, TIM4, AXL	Annexin A5	Liposomes, different viruses	162, 165

RHD, rabbit haemorrhagic disease; HPV, human papiloma virus; AAV, adeno associated virus.

### Size and shape as predictors of VNP and liposome uptake

Experimentally, different labeling strategies contributed to the understanding of particle transport within the body and their uptake by cells of the mononuclear phagocyte system 147. However, each technique applied, might also influence the experimental outcome to a certain degree 147. For instance, fixation techniques required for EM‐based particle tracing, mutation‐based alterations of primary viral sequences to introduce cysteine residues for the attachment of specific labels 148, but also chemical alteration of the particle surface for the covalent attachment of fluorochromes, just to name a few, might all alter the way how particles are being transported, bound, and taken‐up by mononuclear phagocytes, thus respective results obtained with either method should always be confirmed by a second independent method.

Apart from the biophysical limitations of accurately tracing individual particles, it has been clearly shown that particle size represents a decisive factor for free particle transport. In fact, within interstitial fluids only particles (including VNP) with a diameter of <200 nm are freely transported via the lymphatics toward the draining LNs 149. In contrast, for larger particles (diameter of 500–2000 nm) to reach local LNs, uptake and cellular transport by cells belonging to the mononuclear phagocyte system at the site of application of the vaccine depot is required 149, 150. According to one theory, the blind‐ended entrance into lymphatic capillaries controlled by junctions between overlapping endothelial cells and functioning as a molecular sieve, might account for the size‐dependent differences in transport behavior 149. Another theory claims that larger particles might be more firmly trapped within the interstitial space, reducing the likelihood of their entrance into lymphatic vesicles while at the same time increasing the chances for getting phagocytosed by APCs followed by their cell‐based shuttling to the respective sentinel LN 149. Notably, also the shape of nanoparticles may have an influence on uptake kinetics. Studies using artificial nanoparticles showed that larger, disciform particles (nanodiscs) are more efficiently taken up by cells as compared to rod‐like particles, spherical particles, or small nanodiscs 151, 152. This behavior was explained by the increased contact area and thus the stronger adhesive forces between nanodiscs and the respective phagocyte 153. Moreover, nanoparticles with sharp shapes, irrespective of their size, composition, or surface chemistry, may pierce the membranes of endosomes, which contributed to their uptake, much more easily, facilitating, e.g., drug and gene delivery 154.

### Receptors for the uptake of VNP and artificial particles

The main uptake mechanisms of professional APC are macropinocytosis, micropinocytosis, phagocytosis, and receptor‐mediated endocytosis 155. The latter can be strongly enhanced by mannosylation, as shown for the uptake by dendritic cells and macrophages of rabbit hemorrhagic disease virus‐derived VNP 156. Mannosylation of the VNP surface creates ligands for mannose receptors, such as DC‐SIGN, SIGNR1, or CD206, Dectin 1, MR1 expressed on the APC surface 156, contributing to the facilitated engulfment of the respective particles. In the absence of mannosylation, clathrin‐dependent macropinocytosis and phagocytosis represent the most prominent uptake mechanisms for VNP 157. Very similar to rabbit hemorrhagic disease virus particles, also the uptake of adenovirus‐associated particles mainly relies on the clathrin‐dependent mechanisms 158. It is important to emphasize that most of the above cited studies were based on the use of specific inhibitors rather than loss‐of‐function mutations, thus there is still room for refinement of these analyses.

Most interestingly, evidence for direct FcR‐mediated uptake of HPV particles has been shown previously, which involves both low affinity receptors for IgG, i.e., CD16 and CD32 159. Whether this mechanism, which would precede Ig‐mediated VNP uptake is of critical relevance for HPV clearance remains unclear at the moment.

Recently, it was also hypothesized that viruses and consequently VNP might exploit uptake routes, which are usually exploited by exosomes 160, 161. Most mechanistic studies performed in that respect relied on enveloped VNP based on HIV core proteins 161 and revealed that the composition of the lipid envelope itself but not the presence of “bona fide” envelope (viral spike) proteins, determined the mode of up‐take. For both liposomes and VNP alike, the expression of PS on the particle surface might represent an “eat‐me‐signal” for APC. Specifically, PS has been shown to bind to several cell surface receptors on APC, such as TIM1, TIM3, TIM4, or AXL, all capable of initiating VNP uptake, however, depending on the viral backbone via multiple pathways 162. In contrast, GP120‐expressing HIV particles were predominantly taken up by macropinocytosis in an actin dependent manner 163, underlining the importance of the viral envelope proteins in such processes. Similarly, critical contributions to binding and uptake of envelope proteins were also described for hepatitis C‐derived VNPs 164. For liposomes, enhanced PS‐mediated uptake has been proven in human and mouse models of tolerance induction in type I diabetes 165, 166.

Apart from PS‐mediated particle uptake, particle incorporated tetraspanins have been reported to be involved in receptor‐mediated uptake of exosomes 167. A similar mechanism might be used by VNP, which were reported to express tetraspanins such as CD9 CD63 and CD81 in the past 168.

In summary, particle‐based (VNP, liposomes) induction of protective immunity against allergens and/or important effector molecules governing allergic inflammation (Th2 cytokines, IgE or FcεR) represents a versatile method, which might help to complement existing treatment modalities. To extend the full reach of this technology, further refinements as to optimal backbone (virus or liposome) used and co‐signals applied need to be undertaken, which should also include studies precisely defining the particles’ uptake and transport in vivo.

## Conflict of interest

With regards to the authors disclosure of potential conflicts of interest we would like to indicate that W.F.P. holds stocks of Biomay AG and receives honoraria from Novartis and Roche. All other authors have no additional commercial or financial conflict of interest.

## Author contributions

B.K., S.H., M.Z., and W.F.P. wrote the paper.

**Abbreviations**
AAV: adeno‐associated virus
ADA: antidrug antibodies
ADCC: antibody‐dependent cellular cytotoxicity
AIT: allergen‐specific immunotherapy
BM‐DC: BM‐derived DCs
C: cholesterol
CD: cluster of differentiation
CTL: cytotoxic lymphocytes
CuMV: cucumber mosaic virus
DBPC: double blind placebo controlled
DOPE: 1,2‐dioleoyl‐sn‐glycero‐3‐phospha‐tidylethanolamine
FcεR: Fcε receptor
FEV1: forced expiratory volume in 1 second
HBcAg, hepatitis B core antigen
HBsAg: hepatitis B surface antigen
HDM: house dust mite
IFN: interferon
ILC: innate lymphoid cells
LABA: long acting beta antagonists
Mo‐DC: monocyte‐derived dendritic cells
MoMLV: Moloney murine leukemia virus
OH‐Chol: cholesteryl‐3b‐carboxyamidoethy‐lene‐*N*‐hydroxyethylamine
PC: phosphatidylcholine
PS: phosphatidylserine
SPF: specific‐pathogen free
TT: Tetanus‐toxin
VNP: virus‐like nanoparticle
